# Frequency of Acute Adverse Transfusion Reactions at King Saud Medical City, Riyadh, Saudi Arabia

**DOI:** 10.3390/jcm15124432

**Published:** 2026-06-08

**Authors:** Ibrahim Mansour Aboshaya, Muhammed Raihan Sajid, Rimah Abdullah Saleem, Salman Aldosari, Hibba Siraj

**Affiliations:** 1 College of Medicine, Alfaisal University, Riyadh 11533, Saudi Arabia; 2 Pathology and Clinical Laboratory, Transfusion Medicine, King Saud Medical City, Riyadh 12746, Saudi Arabia; 3 Department of Pathology, College of Medicine, Alfaisal University, Riyadh 11533, Saudi Arabia; 4 Department of Biochemistry and Molecular Medicine, College of Medicine, Alfaisal University, Riyadh 11533, Saudi Arabia

**Keywords:** acute transfusion reactions, blood transfusion, adverse event, hemovigilance, allergic reaction, FNHTR, Saudi Arabia, Gulf region

## Abstract

**Background:** Blood transfusion is a life-saving intervention but is associated with adverse reactions in a subset of recipients. An acute transfusion reaction (ATR) is defined as any unfavorable response occurring within 24 h of blood component administration. This retrospective observational study aimed to determine the frequency and characteristics of ATRs at King Saud Medical City (KSMC), Riyadh, Saudi Arabia. **Methods:** We conducted a retrospective study using data from the Transfusion Medicine Laboratory (TML) at KSMC between January 2019 and December 2023. Demographic, clinical, and laboratory data from 238 confirmed ATR records were analyzed using SPSS v21.0. Chi-square, Mann–Whitney U, and risk ratio analyses were performed; *p* < 0.05 was considered statistically significant. **Results:** Among 238 patients (59.2% female; mean age 33.7 ± 18.5 years), allergic reactions were the most frequent ATR (70.2%, *n* = 167), followed by febrile non-hemolytic transfusion reactions (FNHTRs); (24.4%, *n* = 58). Packed red blood cell (PRBC) transfusion was the most commonly implicated product (82.4%; *n* = 196; *p* < 0.001). Fever, rash, itching, and chills were significantly associated with specific reaction subtypes (*p* < 0.001). The pattern of post-transfusion workup differed significantly across reaction types (*p* < 0.001). Age, gender, ward distribution, and reaction timing were not significant predictors of ATR occurrence. **Conclusions:** Allergic reactions are the predominant ATR form at KSMC. Demographic variables are not reliable predictors of ATR. Strengthening hemovigilance systems and clinician education is recommended to improve transfusion safety.

## 1. Introduction

Blood transfusion is an essential clinical intervention for a wide range of medical and surgical conditions, including acute hemorrhage, hematological malignancies, chronic anemia, and operative blood loss [1]. Although blood transfusion is vital for saving lives, each unit transfused carries an inherent risk of adverse reactions and therefore requires rigorous compatibility testing to ensure recipient safety [2]. Standard pre-transfusion protocols include ABO and Rh typing, antibody screening, crossmatch testing, and nucleic acid testing (NAT) for infectious agents such as HIV, HCV, and hepatitis B virus (HBV) surface antigen (HBsAg) [3,4].

### 1.1. Acute Transfusion Reactions: Definition and Clinical Impact

An acute transfusion reaction (ATR) is defined as any clinically significant adverse consequence occurring within 24 h of blood component administration [5,6]. The spectrum of ATRs ranges from mild urticaria and febrile non-hemolytic transfusion reactions (FNHTRs) to life-threatening events, including acute hemolytic transfusion reactions, anaphylaxis, transfusion-related acute lung injury (TRALI), and transfusion-associated circulatory overload (TACO) [7,8]. Global estimates suggest that adverse events occur in a proportion of transfusions [9]. The clinical impact of ATRs extends beyond the immediate episode, contributing to prolonged hospitalization, organ impairment, and increased mortality risk [9,10]. Prompt recognition of early symptoms—fever, chills, urticaria, dyspnea, and hypotension—followed by immediate cessation of the transfusion and systematic post-reaction workup, is critical to optimizing patient outcomes [11].

From a pathophysiological standpoint, ATRs can be classified as immune-mediated or non-immune-mediated. Immune-mediated reactions include allergic and anaphylactic reactions triggered by recipient IgE antibodies against donor plasma proteins, as well as FNHTRs, which arise from cytokine accumulation in stored blood products or from recipient antibody reactions against donor leukocyte antigens [12,13]. Hemolytic reactions occur when ABO-incompatible or alloantibody-mediated destruction of transfused red blood cells activates complement and triggers inflammatory cascades, leading to intravascular hemolysis and, in severe cases, disseminated intravascular coagulation and acute renal failure [14,15]. Non-immune-mediated reactions include TACO, resulting from volume overload, particularly in elderly or cardiac-compromised patients, and transfusion-transmitted infections, which, while rare in screened blood supplies, remain a concern in resource-limited settings [16,17].

### 1.2. International Burden of Transfusion Reactions

A substantial body of international literature has documented the burden of ATRs across diverse healthcare settings. In India, Sharma et al. reported allergic reactions as the most common ATR type, predominantly associated with PRBC and whole blood transfusions [18]. Similarly, Saha et al. identified allergic reactions and FNHTRs as the leading ATR subtypes, with itching and rash as the predominant presenting symptoms [19]. In the United States, Kumar et al. confirmed these patterns, with PRBCs being the most frequently implicated product, and estimated an overall ATR frequency of approximately 6.5 per 1000 transfused units [20]. Hendrickson et al., using multicenter active surveillance across six United States hospitals, found that TRALI, anaphylaxis, and hypotensive reactions were the most severe ATR subtypes, while FNHTRs and allergic reactions were more frequent but clinically milder [21].

In Pakistan, Borhany et al. identified an ATR rate of 0.21% across 35,593 transfused units over five years, with allergic reactions accounting for the majority of cases [22]. A Taiwanese cohort study by Yao et al. found significant correlations between elevated body temperature and moderate ATRs, and between elevated diastolic pressure and severe ATRs, suggesting that physiological parameters at reaction onset carry prognostic value [23]. In sub-Saharan Africa, Gelaw et al. reported that 5.2% of transfused patients in Ethiopia experienced ATRs, with allergic reactions (65%) and FNHTRs (30%) predominating [24]. Muche et al. further demonstrated a 4.13-fold higher ATR risk among patients who did not undergo pre-transfusion compatibility testing [25]. Collectively, these international data establish the global ATR profile and consistently identify allergic reactions and FNHTRs as the most prevalent subtypes, with PRBC transfusion as the most implicated product.

### 1.3. Transfusion Practices and Hemovigilance in Saudi Arabia

Blood transfusion services in Saudi Arabia are coordinated by the Saudi Central Blood Bank (SCBB) under the Ministry of Health, which oversees blood collection, infectious disease testing, storage, and distribution across all regions of the Kingdom. The national blood transfusion protocol follows standards established by the World Health Organization (WHO) and the AABB, ensuring compatibility with international best practices in donor selection, component preparation, and pre-transfusion testing.

Despite the availability of national guidelines, published data on ATRs from Saudi Arabia remain sparse. Most available studies have focused on donor demographics, blood supply management, or seroprevalence of transfusion-transmissible infections, rather than the systematic surveillance of adverse reactions in transfusion recipients. Waggiallah et al. (2021) examined the prevalence of unexpected alloantibodies in Saudi patients prior to transfusion but did not examine post-transfusion reaction outcomes [26]. Zaid and Mustafa (2020) reviewed ABO and Rh blood group distributions across the Middle East and North Africa (MENA) region but did not characterize acute transfusion reactions in clinical settings [27]. The distribution of ABO and Rh blood groups varies substantially across global populations, with the Middle East and North Africa region exhibiting distinct frequency patterns compared to European and East Asian cohorts [27]. To date, no large-scale, multicenter study has systematically characterized the frequency and types of ATRs across Saudi hospitals. This absence of structured hemovigilance data represents a significant gap in national blood safety monitoring.

King Saud Medical City (KSMC) is one of the largest tertiary care referral hospitals in Riyadh, providing services across medical, surgical, obstetric, pediatric, oncological, and critical care specialties. Its high-volume, demographically diverse patient population—including Saudi nationals and expatriates from across the MENA region—makes KSMC an ideal setting for generating baseline epidemiological data on ATRs in the Saudi context.

### 1.4. Hemovigilance in the Gulf Cooperation Council (GCC) and Middle East

The Gulf Cooperation Council (GCC) region—comprising Saudi Arabia, the United Arab Emirates, Kuwait, Qatar, Bahrain, and Oman—has invested substantially in healthcare infrastructure and blood safety over the past two decades [26]. However, published hemovigilance data from the region remain considerably limited compared to North America and Europe, where national reporting systems such as the United States National Healthcare Safety Network (NHSN) and the United Kingdom Serious Hazards of Transfusion (SHOT) scheme have been operational for decades and have generated comprehensive transfusion safety datasets.

In neighboring Iran, Mardani and Balali (2020) analyzed national hemovigilance data and reported transfusion reaction rates of approximately 1.7 per 1000 units transfused, with allergic reactions and FNHTRs as the most common subtypes [28]. However, similar comprehensive analyses are entirely lacking for the Arabian Gulf states, and no unified regional hemovigilance framework currently exists for the GCC. This fragmentation makes cross-national comparisons difficult and limits the capacity for evidence-based quality improvement at a regional level [28]. The present study addresses this gap by providing a detailed single-center analysis of ATRs in Saudi Arabia that can serve as a foundation for future regional collaborative hemovigilance initiatives.

### 1.5. Rationale and Objectives

Given the paucity of baseline ATR data from Saudi Arabia and the GCC region, and the demonstrated importance of hemovigilance in improving transfusion safety outcomes globally, there is a clear and pressing need for epidemiological studies that characterize the frequency, types, and risk factors of acute transfusion reactions in this region. This study aimed to determine the incidence and characteristics of ATRs among patients receiving blood products at KSMC, Riyadh, Saudi Arabia, over a 5-year period (2019–2023). The specific objectives were to: (1) determine the overall frequency and distribution of ATR subtypes; (2) evaluate the association of ATRs with blood component type; (3) identify clinical signs and symptoms significantly associated with specific reaction categories; and (4) explore the pattern of post-transfusion workup across different ATR types.

## 2. Materials and Methods

### 2.1. Study Design and Setting

This observational retrospective study used data retrieved from the Transfusion Medicine Laboratory (TML) at the Blood Bank of King Saud Medical City (KSMC), Riyadh, Saudi Arabia. KSMC is a 1500-bed Ministry of Health tertiary care hospital serving as a major referral center for Riyadh and the surrounding regions. Records spanning January 2019 to December 2023 were reviewed.

### 2.2. Sample Size and Eligibility Criteria

Convenience sampling was employed. A total of 238 confirmed ATR records were identified over the five-year study period. All patients diagnosed with an ATR during the study period were eligible for inclusion regardless of age, gender, diagnosis, or ward of admission. In cases where the same patient experienced multiple distinct ATR events during separate transfusion episodes, each event was recorded as an individual case. Patients with chronic or delayed transfusion reactions (defined as reactions occurring more than 24 h post-transfusion) and those with incomplete records lacking key variables—including compatibility testing results or symptom documentation—were excluded from the analysis.

### 2.3. Data Collection

Data were extracted from medical records and transfusion cards maintained at the blood bank. Collected variables included patient demographics (age, gender, ward of admission), ABO and Rh blood grouping, transfusion product type (PRBC, platelets [PLT], apheresis platelets [APLTs], and fresh frozen plasma [FFP]), pre- and post-transfusion compatibility testing results, reaction type, and clinical signs and symptoms at the time of reaction.

All ATR cases were identified through the hemovigilance system at KSMC, wherein suspected reactions were initially reported by bedside nurses or physicians to the Transfusion Medicine Laboratory. Subsequently, transfusion medicine physicians reviewed each reported event, performed systematic clinical and laboratory evaluation, and rendered a final reaction classification based on standardized diagnostic criteria (detailed below). Laboratory confirmation included post-transfusion direct antiglobulin testing, repeat ABO/Rh typing of post-transfusion blood samples, visual inspection of plasma for hemolysis, and, when clinically indicated, Gram staining and culture of residual blood product units.

Reaction types were classified according to standardized criteria adapted from the AABB (American Association of Blood Banks) Technical Manual definitions. Allergic reactions were defined by the presence of urticaria, pruritus, flushing, or rash occurring within 4 h of transfusion initiation in the absence of fever. FNHTR was defined as a temperature rise of ≥1 °C or 2 °F from pre-transfusion baseline or new-onset fever ≥ 38 °C (100.4 °F) occurring during or within 4 h of transfusion completion, without evidence of hemolysis or bacterial contamination. TACO was defined as new-onset or worsening respiratory distress with evidence of pulmonary edema on physical examination or chest imaging occurring within 6 h of transfusion. TRALI was defined as new-onset acute lung injury occurring within 6 h of transfusion, with hypoxemia (PaO_2_/FiO_2_ ≤ 300 mmHg or SpO_2_ < 90% on room air) and bilateral pulmonary infiltrates without evidence of hydrostatic pulmonary edema or alternative risk factors for acute lung injury. Undetermined reactions were those with insufficient clinical or laboratory data to assign a specific category.

Reactions classified as ‘undetermined’ (*n* = 8, 3.4%) were those in which: (a) symptoms were temporally associated with transfusion but did not meet full diagnostic criteria for any specific ATR subtype; (b) laboratory workup was incomplete due to insufficient sample volume or specimen hemolysis; or (c) competing clinical conditions (e.g., underlying sepsis, medication reactions) could not be confidently excluded as the etiology of the observed symptoms.

### 2.4. Ethical Considerations

Institutional Review Board (IRB) approval was obtained from the Research Center, King Saud Medical City, Riyadh, Saudi Arabia (Reference: H1RI-17-DEC23-01; approval date: 20 December 2023). The study was conducted in accordance with the Declaration of Helsinki. All patient data were de-identified prior to analysis, and records were stored securely to ensure confidentiality. As a retrospective study using anonymized routinely collected clinical data, the requirement for individual patient informed consent was waived by the IRB.

### 2.5. Statistical Analysis

Data were coded and entered into Microsoft Excel and subsequently analyzed using SPSS v21.0 (IBM Corp., Armonk, NY, USA). Continuous variables were expressed as mean ± standard deviation (SD) or median (interquartile range, IQR) as appropriate. The Kolmogorov–Smirnov test was used to assess the normality of distribution. Normally distributed variables were compared between groups using Student’s *t*-test or one-way ANOVA; non-normally distributed variables were compared using the Mann–Whitney U test or the Kruskal–Wallis H-test. Categorical associations were analyzed using the chi-square test. All statistical tests were two-tailed, and statistical significance was set at *p* < 0.05.

Rationale for risk ratio analysis: Risk ratios were calculated to quantify the association between categorical predictor variables (product type, gender, ward) and the outcome of allergic reaction (versus non-allergic reaction). This approach was chosen because allergic reactions comprised the majority of ATRs (70.2%), making a binary outcome (allergic vs. non-allergic) statistically tractable while preserving clinical interpretability.

Multivariable analysis: Multivariable logistic regression was considered to control for potential confounding between variables (e.g., product type and ward, or age and product type). However, preliminary model diagnostics revealed complete separation for several predictor variables (particularly product type, where FFP and PLT were perfectly associated with allergic reactions) and limited sample size (*n* = 238) relative to the number of potential confounders. Consequently, multivariable models would have produced unstable estimates with extremely wide confidence intervals, consistent with the small subgroup sizes observed in univariable analyses (e.g., FFP RR: 15.1, 95% CI: 2.1–107.8). 

Post hoc power analysis: Post hoc power analysis was conducted using GPower 3.1. Assuming a two-tailed alpha of 0.05 and the observed effect size for FFP versus PRBC (Cramér’s V = 0.28), the achieved power for the comparison of allergic versus non-allergic reactions across product types was 0.89, indicating adequate power for this primary comparison. However, comparisons involving subgroups with cell counts < 5 (e.g., APLT) were underpowered (observed power < 0.30), and these risk ratio estimates should be interpreted with caution.

## 3. Results

### 3.1. Demographic Characteristics

A total of 238 patient ATR records were analyzed from January 2019 to December 2023. The majority of patients were female (141, 59.2%), with a mean age of 33.2 ± 17.1 years among females and 34.5 ± 20.4 years among males (overall mean 33.7 ± 18.5 years; range 3–82 years). Female preponderance was most pronounced in the 21–30 and 31–40 age groups, consistent with the high volume of obstetric admissions at KSMC. The distribution of ATR cases across age groups and gender was statistically significant (chi-square = 24.7, *p* = 0.001), though the difference in mean age between males and females was not statistically significant (Mann–Whitney U; Z = 0.6, *p* = 0.556). The total number of blood units transfused at KSMC during the study period was not available from the electronic records system at the time of data extraction, precluding calculation of absolute ATR rates per unit transfused or per transfused patient. Thus, the 238 cases represent the identified ATR burden but cannot be expressed as a true incidence rate. The full demographic distribution is presented in Table 1.

### 3.2. Distribution of Acute Transfusion Reaction Types

Allergic reactions were the most frequent ATR subtype, occurring in 167 patients (70.2%), followed by FNHTRs in 58 patients (24.4%). The remaining reaction types—TACO (*n* = 4, 1.7%), TRALI (*n* = 1, 0.4%), and undetermined (*n* = 8, 3.4%)—were grouped as “Other” for subsequent comparative analyses due to their low individual frequencies. Table 2 presents the full distribution of reaction types.

### 3.3. Association with Sociodemographic Characteristics

No statistically significant association was found between ATR type and gender (*p* = 0.753) or ward of admission (*p* = 0.501). Females represented 59.2% of ATR cases across all three reaction groups. Emergency room (ER) patients constituted the largest ward subgroup (78, 32.8%), followed by general ward patients (53, 22.3%), intensive care unit (ICU) patients (35, 14.7%), maternity ward patients (34, 14.3%), oncology patients (19, 8.0%), and pediatric patients (19, 8.0%). Table 3 provides the full sociodemographic breakdown by ATR type.

### 3.4. Association with Blood Product Type and Post-Transfusion Workup

PRBC transfusion was the blood product most associated with ATRs (196, 82.4%), with a highly significant association across all reaction types (*p* < 0.001). FFP was the second most implicated product among allergic reactions (28, 16.8%), followed by PLTs (12, 7.2%) and APLTs (1, 0.4%). With respect to post-transfusion workup, compatibility testing alone was performed in 99.4% of allergic reaction cases and 100% of “Other” reaction cases, whereas compatibility testing combined with microbiological culture was performed in 69.0% of FNHTR cases—reflecting clinical suspicion of a bacteremic component in febrile reactions. This difference in workup approach across reaction types was highly significant (*p* < 0.001). Full data are presented in Table 4.

### 3.5. Clinical Signs and Symptoms

Among the 238 cases, itching (77, 32.4%), fever (60, 25.2%), rash (39, 16.4%), chills (4, 1.7%), and chest pain (2, 0.8%) were each significantly associated with specific ATR subtypes (all *p* ≤ 0.001). Itching and rash were confined to the allergic reaction group, while fever was observed exclusively in FNHTR cases, reflecting distinct immunological mechanisms underlying each reaction type. Tachypnea was reported in 14 cases (5.9%), all within the allergic group, though this did not reach statistical significance (*p* = 0.593). Other symptoms—including cough, headache, hypertension, anxiety, urticaria, swelling, and general body pain—did not demonstrate a statistically significant association with ATR type (all *p* > 0.05). Table 5 presents the complete symptom distribution by reaction type.

### 3.6. Association of Demographic and Product-Related Variables with Allergic Transfusion Reactions

Risk ratios were calculated to assess associations with allergic reactions (Table 6). Blood product type was the strongest predictor: FFP (risk ratio [RR] = 15.1; 95% CI: 2.1–107.8; *p* = 0.001) and PLT (RR = 13.6; 95% CI: 1.8–103.2; *p* = 0.005) were associated with significantly higher allergic reaction rates compared to PRBC. PRBCs (RR = 0.81; *p* = 0.285) and APLTs (apheresis platelets) (RR = 1.02; *p* = 0.999) were not significantly different from PRBC. No significant associations were observed for age (*p* = 0.108), gender (*p* = 0.800), or ward of admission (global *p* = 0.208). Note: Analyses involving cells with *n* < 5 (APLT row) should be interpreted with caution.

## 4. Discussion

### 4.1. Principal Findings

This five-year retrospective study of 238 confirmed ATRs at King Saud Medical City (KSMC), Riyadh, Saudi Arabia, yielded three principal findings. First, allergic reactions were the most common ATR subtype (70.2%), followed by FNHTRs (24.4%). Second, PRBC transfusion was the most frequently associated blood product with ATRs (82.4%; *p* < 0.001). Third, the pattern of post-transfusion workup differed significantly across reaction types (*p* < 0.001), with FNHTR cases more frequently undergoing microbiological culture in addition to compatibility testing, while demographic variables—age, gender, ward distribution, and reaction timing—were not significant predictors. These findings provide the first detailed characterization of ATRs from a large tertiary care hospital in Saudi Arabia, addressing a significant gap in the regional hemovigilance literature.

### 4.2. Demographic Characteristics and Regional Context

The mean age of patients in this study was 33.7 ± 18.5 years, which is younger than the median age of 46 years reported by Sahu and Bajpai in a specialized liver center cohort [9] and substantially younger than the mean of 67 years observed by Kumar et al. in a United States hospitalized population [20]. These differences likely reflect variations in institutional case mix and the demographic profile of the Saudi population, which has a relatively young age structure compared to Western countries. The high volume of maternity services at KSMC is another contributing factor, as obstetric patients account for a significant proportion of transfusion recipients at this institution.

Females comprised 59.2% of all ATR cases, with a preponderance in the 21–40 age group. This finding is plausibly attributable to obstetric blood loss and pregnancy-related anemia—conditions that are prevalent among this age group and necessitate transfusion in a significant proportion of patients. Similar female predominance and a younger age distribution have been reported in studies from neighbouring countries, Pakistan and Ethiopia, suggesting that obstetric transfusion indications may be a major driver of ATR demographics across low- and middle-income healthcare settings [22,24].

### 4.3. Frequency and Types of Acute Transfusion Reactions

Allergic reactions accounted for 70.2% of all ATRs, which is consistent with reports from Ethiopia (65%) [24] and India [18,19], where allergic reactions similarly predominate. The pathophysiology of allergic transfusion reactions involves IgE-mediated sensitization to donor plasma proteins, which, upon re-exposure, triggers mast cell degranulation and histamine release, resulting in urticaria, pruritus, and, in severe cases, anaphylaxis [12]. The FNHTR was the second most prevalent subtype (24.4%), arising from recipient antibodies directed against donor leukocyte antigens or from the release of pyrogenic cytokines such as IL-1, IL-6, and TNF-α from leukocytes during storage [13,21].

In contrast, severe reactions—TRALI and TACO—were rare, each accounting for less than 2% of cases. The low frequency of TRALI and TACO likely reflects both their true rarity in a non-critical-care-predominant population and the potential for underreporting inherent in passive hemovigilance systems [21]. Hendrickson et al., using active multicentre surveillance, identified TRALI and anaphylaxis as the predominant severe reactions, with higher frequency rates than those observed in passive reporting studies [21]. This discrepancy underscores the importance of active surveillance methodology in capturing rare but clinically significant events.

### 4.4. Blood Products Associated with Transfusion Reactions

PRBC transfusion was most commonly associated with ATRs (82.4%), consistent with Kumar et al. [20]. The predominance of PRBCs reflects their high clinical utilization volume across all ward types rather than an intrinsically higher immunogenicity per unit. PRBC are the most transfused blood component globally and therefore represent the greatest absolute exposure to transfusion-related risks. Interestingly, FFP and PLTs—products with high plasma protein content—were disproportionately represented among allergic reactions, accounting for 16.8% and 7.2% of allergic ATRs, respectively. This is consistent with the known immunogenicity of plasma proteins, which can elicit IgE-mediated allergic responses in sensitized recipients [12]. The differential reaction profiles across blood component types reinforce the need for component-specific surveillance, risk communication, and mitigation strategies such as washing or irradiation for high-risk patients.

### 4.5. Predictors of Acute Transfusion Reactions

No significant associations were observed between ATR type and age (*p* = 0.713), gender (*p* = 0.753), or ward distribution (*p* = 0.501). These findings are consistent with Kar et al., who reported no significant sex-based difference in ATR distribution (*p* = 0.762) [29], and align with findings from Bazaali, who identified clinical variables—including prior transfusion history and sickle cell disease—rather than demographic factors as the primary ATR risk variables [30]. Risk ratio analysis confirmed that blood product type was the strongest predictor of allergic reactions: FFP (RR = 15.1; *p* = 0.001) and PLTs (RR = 13.6; *p* = 0.005) were associated with significantly higher allergic reaction rates compared to PRBC, while age, gender, and ward distribution showed no significant associations. Our findings suggest that in this cohort, demographic characteristics did not reliably distinguish ATR types, and that clinical assessment should focus on transfusion history, underlying hematologic conditions, and appropriate product selection.

### 4.6. Association of Post-Transfusion Workup with Reaction Types

Our data demonstrates an association between the type of post-transfusion workup and the category of ATR experienced. Among patients in this cohort, those with allergic reactions were nearly universally (99.4%) managed with compatibility testing alone, whereas a substantial proportion of FNHTR cases (69.0%) underwent additional microbiological culture. This finding likely reflects clinical decision-making: febrile reactions prompting concern for bacterial contamination, triggering additional diagnostic evaluation. However, it is critical to note that without a control group of transfused patients without ATRs and given that all patients in this cohort received some form of post-transfusion evaluation, we cannot conclude that compatibility testing exerts a ‘protective effect’ or that it independently reduces ATR risk. The pattern of post-reaction workup differs significantly between reaction types, consistent with established clinical algorithms [31]. These findings are consistent with current standards requiring universal pre-transfusion compatibility testing, as the association observed reflects appropriate diagnostic evaluation rather than a causal protective effect.

### 4.7. Clinical Signs and Symptoms as Diagnostic Markers

The strong specificity of symptom–reaction relationships observed in this study is pathophysiologically coherent and of direct clinical utility. The exclusive confinement of itching and rash to the allergic reaction group, and of fever to FNHTR cases, reflects the fundamentally distinct immunological mechanisms underlying these two reaction categories. These findings support the use of symptom-based algorithms for rapid ATR classification at the bedside, enabling timely and appropriate management. Yao et al. similarly reported that chills, rash, itching, and fever are the most reliable clinical determinants for identifying and prognosticating ATR events [23]. Afroz et al. identified rash, itching, and fever/chills as the three most frequently reported symptoms in ATR cases across their five-year hemovigilance study [32]. Callera et al. and Lubart et al. similarly reported that allergic reactions and FNHTRs are the most common ATR subtypes across diverse populations [33,34]. The convergence of these findings across geographically diverse settings—Taiwan, Bangladesh, Brazil, Israel, and Saudi Arabia—supports the generalizability of symptom-based ATR recognition tools and argues for their incorporation into routine transfusion medicine training programs.

### 4.8. Study Limitations

Several limitations of this study must be acknowledged. First, the retrospective, single-center design limits the generalizability of findings to other healthcare settings within Saudi Arabia and the broader GCC region. Second, there may have been underreporting of mild reactions—particularly mild allergic reactions that resolved spontaneously without transfusion cessation and did not prompt a formal report to the Transfusion Medicine Laboratory. Third, information bias may have arisen from incomplete or inconsistent documentation in the medical record, including missing symptom descriptions, incomplete laboratory workup, and variability in the level of detail recorded by different clinicians. Fourth, data on the total number of units transfused during the study period were not available from the institutional electronic health record system at the time of data extraction, precluding calculation of an absolute ATR rate per unit transfused or per transfused patient. Fifth, we were unable to adjust for patient comorbidities (e.g., history of atopy, prior transfusion history, underlying hematologic malignancy, immunocompromised status) that may confound the association between product type and reaction risk. Sixth, we did not systematically grade ATR severity (mild, moderate, severe, or fatal) using standardized scales such as the International Society of Blood Transfusion (ISBT) severity grading, limiting our ability to describe the clinical impact beyond reaction type. Seventh, the small sample sizes for certain product categories (particularly APLT, *n* = 1; TACO, *n* = 4; TRALI, *n* = 1) resulted in wide confidence intervals for risk ratio estimates involving these subgroups. These estimates should be interpreted as hypothesis-generating rather than definitive.

### 4.9. Implications for Clinical Practice and National Hemovigilance

Comparison with established international hemovigilance systems: The United Kingdom’s SHOT (Serious Hazards of Transfusion) scheme, established in 1996, represents the world’s longest-running national hemovigilance system. SHOT operates as an independent, professionally led reporting system that has achieved reporting rates exceeding 90% of hospitals and has driven measurable reductions in transfusion-related mortality through evidence-based recommendations. The US National Healthcare Safety Network (NHSN) Hemovigilance Module, launched in 2010, operates through the CDC and mandates reporting of transfusion-related fatalities, with voluntary reporting of non-fatal reactions. European Union hemovigilance, governed by Directive 2002/98/EC, requires all member states to maintain national reporting systems with standardized definitions. The present study’s reliance on passive surveillance—wherein reactions are reported at the discretion of clinical staff without systematic case-finding—likely underestimates the true ATR burden, particularly for milder reactions that resolve without transfusion cessation and for delayed-onset reactions.

Active versus passive surveillance at KSMC: KSMC currently operates a passive hemovigilance system, with case identification dependent on clinician-initiated reports to the Transfusion Medicine Laboratory. Active surveillance—involving prospective screening of all transfused patients, daily chart review, and systematic laboratory testing—has been shown to detect 3- to 5-fold higher ATR rates compared to passive reporting in comparable hospital settings. The transition to active surveillance would require additional personnel, dedicated hemovigilance nursing staff, and integration with the electronic health record for automated trigger tools.

The findings of this study have several practical and policy-level implications. First, universal pre-transfusion compatibility testing should be strictly enforced across all clinical settings at KSMC and across Saudi Arabia, as recommended by current international standards. Second, clinician education programs should emphasize the diagnostic value of specific symptoms—fever, itching, rash, and chills—in identifying ATR subtypes and guiding management decisions and should address the importance of prompt reporting of suspected reactions. Third, institutions should consider transitioning from passive to active ATR surveillance to capture the true burden of reactions, particularly rare severe events such as TRALI and TACO that are likely underrepresented in passive reporting systems [10].

The current study provides a preliminary dataset that could contribute to the development of a national framework for hemovigilance. Future prospective multicenter studies across the GCC, incorporating active surveillance methodology and standardized reaction definitions, are strongly recommended to build on these findings.

## 5. Conclusions

In this five-year retrospective study at King Saud Medical City, Riyadh, allergic reactions were the most prevalent ATR subtype (70.2%), followed by FNHTRs (24.4%). PRBC transfusion was the most implicated blood product (82.4%). Demographic variables—including age, gender, ward distribution, and reaction timing—were not significant predictors of ATR occurrence or type. The pattern of post-transfusion workup differed significantly across reaction types, with FNHTR cases more frequently undergoing microbiological culture in addition to compatibility testing. Fever, itching, rash, and chills were the most diagnostically informative clinical features, each significantly and specifically associated with distinct ATR categories.

These findings provide the first systematic characterization of ATR epidemiology from a large Saudi tertiary care hospital and highlight critical areas for improvement in transfusion safety practice. The results strongly support universal pre-transfusion compatibility testing, systematic symptom-based ATR monitoring, and the development of a national hemovigilance infrastructure in Saudi Arabia. Prospective multicenter studies across the Gulf Cooperation Council region are recommended to establish a comprehensive and representative ATR profile for this underrepresented healthcare context.

## Figures and Tables

**Table 1:** Age and gender distribution of the 238 patients with acute transfusion reactions.

Age (Years)	Female *n* (%)	Male *n* (%)	Total *n* (%)
≤10	13 (9.2%)	14 (14.4%)	27 (11.3%)
11–20	15 (10.6%)	14 (14.4%)	29 (12.2%)
21–30	30 (21.3%)	15 (15.5%)	45 (18.9%)
31–40	52 (36.9%)	16 (16.5%)	68 (28.6%)
41–50	18 (12.8%)	15 (15.5%)	33 (13.9%)
51–60	3 (2.1%)	10 (10.3%)	13 (5.5%)
61–70	3 (2.1%)	9 (9.3%)	12 (5.0%)
>70	7 (5.0%)	4 (4.1%)	11 (4.6%)
Total	141 (59.2%)	97 (40.8%)	238 (100%)
Mean ± SD	33.2 ± 17.1	34.5 ± 20.4	33.7 ± 18.5
*p* (Chi-square)	Chi-sq = 24.7; *p* = 0.001		
*p* (Mann–Whitney U)	–	–	Z = 0.6; *p* = 0.556

**Table 2:** Frequency distribution of acute transfusion reaction types observed (*N* = 238).

Reaction Type	*n* (%)
Allergic	167 (70.2%)
Febrile Non-Hemolytic Transfusion Reaction (FNHTR)	58 (24.4%)
Undetermined	8 (3.4%)
Transfusion-Associated Circulatory Overload (TACO)	4 (1.7%)
Transfusion-Related Acute Lung Injury (TRALI)	1 (0.4%)
Total	238 (100%)

**Table 3:** Distribution of acute transfusion reaction type by sociodemographic characteristics.

Characteristic	Description	Allergic *n* (%)	FNHTR *n* (%)	Others *n* (%)	Total *n* (%)	*p*
Gender	Female	98 (58.7%)	34 (58.6%)	9 (69.2%)	141 (59.2%)	0.753
	Male	69 (41.3%)	24 (41.4%)	4 (30.8%)	97 (40.8%)	
Ward	General	32 (19.2%)	16 (27.6%)	5 (38.5%)	53 (22.3%)	0.501
	Maternity	27 (16.2%)	6 (10.3%)	1 (7.7%)	34 (14.3%)	
	ER	51 (30.5%)	23 (39.7%)	4 (30.8%)	78 (32.8%)	
	Oncology	14 (8.4%)	3 (5.2%)	2 (15.4%)	19 (8.0%)	
	ICU	27 (16.2%)	7 (12.1%)	1 (7.7%)	35 (14.7%)	
	Pediatric	16 (9.6%)	3 (5.2%)	0 (0.0%)	19 (8.0%)	

**Table 4:** Distribution of acute transfusion reaction type by blood product type and post-transfusion workup.

Characteristic	Description	Allergic *n* (%)	FNHTR *n* (%)	Others *n* (%)	Total *n* (%)	*p*
Product	APLT	1 (0.6%)	0 (0.0%)	0 (0.0%)	1 (0.4%)	<0.001
	FFP	28 (16.8%)	0 (0.0%)	1 (7.7%)	29 (12.2%)	
	PLT	12 (7.2%)	0 (0.0%)	0 (0.0%)	12 (5.0%)	
	PRBC	126 (75.4%)	58 (100%)	12 (92.3%)	196 (82.4%)	
Post-Tx Workup	Compatibility testing	166 (99.4%)	18 (31.0%)	13 (100%)	197 (82.8%)	<0.001
	Compatibility testing + Culture	1 (0.6%)	40 (69.0%)	0 (0.0%)	41 (17.2%)	

**Table 5:** Distribution of acute transfusion reaction type by clinical signs and symptoms.

Symptom	Allergic *n* (%)	FNHTR *n* (%)	Others *n* (%)	Total *n* (%)	*p*
Chills	0 (0.0%)	0 (0.0%)	4 (30.8%)	4 (1.7%)	0.001
Chest pain	0 (0.0%)	0 (0.0%)	2 (15.4%)	2 (0.8%)	<0.001
Rash	37 (22.2%)	0 (0.0%)	2 (15.4%)	39 (16.4%)	<0.001
Itching	76 (45.5%)	0 (0.0%)	1 (7.7%)	77 (32.4%)	<0.001
Fever	0 (0.0%)	58 (100%)	2 (15.4%)	60 (25.2%)	<0.001
Cough	4 (2.4%)	0 (0.0%)	0 (0.0%)	4 (1.7%)	0.661
Headache/Hypertension/Anxiety	5 (3.0%)	2 (3.4%)	1 (7.7%)	8 (3.4%)	0.499
Urticaria	5 (3.0%)	0 (0.0%)	0 (0.0%)	5 (2.1%)	0.496
Tachypnea	14 (8.4%)	0 (0.0%)	0 (0.0%)	14 (5.9%)	0.593
Swelling	6 (3.6%)	0 (0.0%)	0 (0.0%)	6 (2.5%)	0.785
General body pain	5 (3.0%)	0 (0.0%)	0 (0.0%)	5 (2.1%)	0.496

**Table 6:** Association of demographic and product-related variables with allergic transfusion reactions (*N* = 238).

Variable	Category	Allergic (*n* = 167)	Non-Allergic * (*n* = 71)	Risk Ratio (95% CI)	*p*-Value †
Product					
	PRBC (ref)	126 (75.4%)	70 (98.6%)	1	—
	FFP	28 (16.8%)	1 (1.4%)	11.9 (1.7–83.4)	0.001
	PLT	12 (7.2%)	0 (0.0%)	10.7 (1.4–81.2)	0.005
	APLT	1 (0.6%)	0 (0.0%)	1.02 (0.98–1.07)	0.999
Age (years)	Mean ± SD	32.5 ± 18.0	36.6 ± 19.5	MD: −4.1 (−9.1 to +0.9) ‡	0.108
Gender	Female	98 (58.7%)	43 (60.6%)	1	0.8
	Male	69 (41.3%)	28 (39.4%)	1.04 (0.76–1.42)	
Ward	General	32 (19.2%)	21 (29.6%)	1	0.208 §
	Maternity	27 (16.2%)	7 (9.9%)	1.38 (0.91–2.08)	
	ER	51 (30.5%)	27 (38.0%)	0.90 (0.64–1.27)	
	Oncology	14 (8.4%)	5 (7.0%)	1.04 (0.64–1.69)	
	ICU	27 (16.2%)	8 (11.3%)	1.18 (0.81–1.71)	
	Pediatric	16 (9.6%)	3 (4.2%)	1.38 (0.90–2.11)	

* Non-Allergic group includes FNHTR (*n* = 58), TACO (*n* = 4), TRALI (*n* = 1), and Undetermined (*n* = 8). † Fisher’s exact test or chi-square test for categorical variables; Mann–Whitney U test for age. ‡ Mean difference (Allergic—Non-Allergic). § Global *p*-value for ward variable (chi-square = 8.65, df = 5).

## Data Availability

The data supporting the findings of this study are available from the corresponding author upon reasonable request, subject to institutional data-sharing policies.
